# Deictic Navigation Network: Linguistic Viewpoint Disturbances in Schizophrenia

**DOI:** 10.3389/fpsyg.2019.01616

**Published:** 2019-07-24

**Authors:** Linde van Schuppen, Kobie van Krieken, José Sanders

**Affiliations:** Centre for Language Studies, Radboud University, Nijmegen, Netherlands

**Keywords:** deixis, interaction, intersubjectivity, language, origo, perspective, schizophrenia, subjectivity

## Abstract

This paper introduces the Deictic Navigation Network, a cognitive-linguistic framework to analyze and clarify the nature of viewpoint disturbances in language, applied to schizophrenia. We argue that such disturbances have linguistic counterparts in the use of deixis: linguistic elements of which the interpretation relies on the situational context of the discourse and their connection to a subject-bound perspective. The DNN connects such linguistic phenomena to three viewpoint disturbances, which can manifest in different degrees of extremity: (i) the reduced capacity to recognize one’s own subjective perspective and the subjective perspectives of others; (ii) the reduced capacity to separate present perspectives from distinct past, future, and hypothetical perspectives; and (iii) the reduced capacity to integrate projected viewpoint structures into the actual here-and-now. We explain how application of the DNN to language in schizophrenia enables the localization of perspectivization disturbances and helps to clarify the nature of disturbances in the ability to build complex viewpoint structures in language as well as cognition.

## Introduction

Schizophrenia is commonly conceptualized as a so-called “self-disorder” (Mishara et al., 2013), meaning that the unifying principle that lies at the basis of schizophrenic symptoms is a disturbance of the “bodily,” or “minimal” self (Gallese and Ferri, 2014; Zahavi, 2014). A profound disruption of the structures of subjectivity itself, connected to a fundamental alienation from one’s body, might underlie distinct schizophrenic phenomena, like self-demarcation problems and the experience of “losing one’s self” and, by extension, difficulties in managing boundaries between one’s self, the world and others (Sass, 1994). In aiming to comprehend these schizophrenic phenomena “from the inside,” researchers across disciplines (linguistics, psychiatry, and cognitive philosophy) focus on language use, considering that language can be seen as a window into the mind (Lysaker et al., 2002; Buck and Penn, 2015; Demjén and Semino, 2015; Minor et al., 2015). Studies in this domain have examined various linguistic categories, such as pronoun use (Buck and Penn, 2015; Hong et al., 2015; Fineberg et al., 2016) and coherence markers (Saavedra, 2010; Allé et al., 2015; Bedi et al., 2015; Willits et al., 2018). Although these studies have revealed patterns in language use that appear typical to schizophrenia patients, it is yet unclear whether and how these linguistic patterns might correspond to as well as elucidate cognitive deficits associated with the disorder, in particular disturbances in the ability to recognize and distinguish subject-bound perspectives (Corcoran et al., 1997; Langdon and Ward, 2009; Fuchs, 2015; Van Duppen, 2016).

This paper presents an analytical framework that enables researchers to precisely point out specific cognitive viewpoint disturbances in schizophrenia through the analysis of language, specifically through linguistic markers of *perspective* which express subjects’ position in relation to others, the world and themselves (Dancygier and Sweetser, 2012; Igl and Zeman, 2016; Van Krieken et al., 2017). Analyses employing this framework have the potential to advance our understanding of the exact nature of viewpoint and intersubjectivity disturbances in schizophrenia. Such analyses can furthermore inform debates on the nature of Theory of Mind (ToM)^1^ in schizophrenia patients, which comprises the ability to recognize the minds of others and to (unconsciously) reason about and anticipate their beliefs, intentions, and desires. Empirical studies have demonstrated ToM disturbances in schizophrenia patients, connecting them to various prominent psychotic symptoms like delusions and thought disorder (Pickup and Frith, 2001; Langdon and Ward, 2009; Brüne, 2018), although the exact nature of these disturbances is still debated (Cardella, 2018, p. 53–63). These deficits seem to be especially prominent in pragmatic comprehension and production of speech, such as managing direct speech acts, irony and deceits (Parola et al., 2018). We propose to turn to an analysis of the language use of patients to shed more light on theories on intersubjectivity and self-disturbance, by connecting linguistic phenomena to three cognitive requirements for perspective-taking.

## Perspective and Origo

**FIGURE 1:**
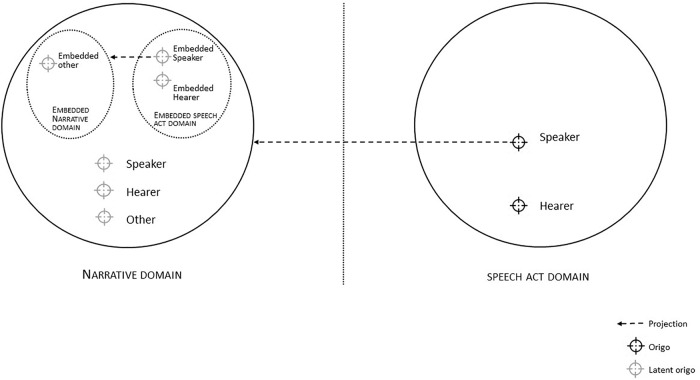
Deictic Navigation Network with a Speech Act Domain representing the origos of Speaker and Hearer in the present and a Narrative Domain representing the origos of subjects that the discourse is about.

Our point of departure is that cognitive deficits in recognizing and distinguishing perspectives parallel linguistic expressions of perspective in intersubjective discourse.^2^ To describe and analyze such discourse adequately, a general model is required of how perspectives are construed and navigated in language and cognition. We introduce a Deictic Navigation Network that is intended to do just that. This framework enables analyses of viewpoint phenomena in natural discourse by accounting for two fundamental principles: first, that natural discourse is multi-viewpointed, and, second, that natural discourse is characterized by recursive patterns (Sanders et al., 2012). The model is visualized in Figure 1 and will be introduced below.

The DNN can be seen as a model for intersubjective communication that represents the multitude of perspectives which Speaker and Hearer need to represent cognitively – albeit minimally (Vesper et al., 2010; Butterfill and Apperly, 2013) – and manage linguistically. This includes the perspectives of Speaker and Hearer in the Speech Act Domain, which represents the communicative process in which the discourse is established here-and-now, as well as the perspectives of subjects that the discourse is *about* in the Narrative Domain.

Figure 1 depicts the *origo* as unifying principle of cognitive and linguistic perspective taking and navigation, that is, the vantage point of the speaking subject (Langacker, 1985). The *origo* (Bühler, 1934/1982) represents the subject in its immediate here-and-now and is to be understood as “the origin of a coordinate system of “subjective orientation” (Fricke, 2002, p. 208); (Bühler, 1934/1982, p. 102). All perceptual and speech acts originate in, and are anchored to, this origo, since these acts are always performed by a body that can only be situated at one place at one time. When two subjects verbally interact, the Speaker needs to consider the origo of the Hearer, in addition to her own, and that of any other actor that figures in the conversation, and acknowledge that the multitude of origos corresponds to a multitude of coordinate systems, e.g.: what counts as *here* and *there* is origo-dependent and might differ between Speaker, Hearer and Others, who all have a different bodily orientation. The notion of origo can help us understand the structure of subjective experiences and how these experiences are expressed in language (Dancygier and Sweetser, 2012; Zeman, 2017).

Successful interaction is a process in which two individuals take turns as Speaker and Hearer in building and modifying a common ground that forms the basis for mutual understanding (Clark and Brennan, 1991; Clark, 1996). This common ground dynamically encompasses the knowledge that is shared between people *in a specific interaction* (“Are you coming to *the party*?”). Each interaction revolves around such a common ground that is built upon the accumulating utterances in the discourse and feeds upon general knowledge, culturally constituted schemata and know-how about the world, language, and social conventions (Sanders et al., 2012). Discourse participants not only retract information from their common ground, but modify it during interaction by adding information, correcting for misunderstandings and checking whether all participants are on the same page.

Crucially, discourse participants must be able to navigate both actual and virtual origos in interaction, to fully integrate text and context in their language comprehension; this requires the cognitive and pragmatic abilities to use deixis in keeping track in the origos representation (Sperber and Wilson, 1986/1995). In the first place, such navigation requirements apply to the origos of Speaker and Hearer that are represented in the Speech Act Domain, which depicts the here-and-now of the conversational act. Apart from these physical origos, a potentially infinite number of latent origos can be assumed in each discourse. These are the origos of subjects that the discourse is *about*. These are depicted in the Narrative Domain. This domain roughly corresponds to what is known as the *situation model* or *mental model* which people mentally construct when producing and processing a narrative and which they continuously update and modify as the narrative unfolds in time and space (Zwaan et al., 1995; Zwaan and Radvansky, 1998). Research within this situation model framework has shown that people mentally simulate and may even embody the perspectives of narrative characters (e.g., Yaxley and Zwaan, 2007; Brunyé et al., 2011), indicating that people are indeed capable of representing multiple origos simultaneously and of temporarily projecting their own origo onto that of others in order to understand scenarios from a perspective that is not their own present perspective.

In the DNN, the Narrative Domain is conceptualized as including all references to events and situations that do not take place in the immediate here-and-now of the Speech Act Domain. Narrating what happened in the past or could happen in the future, as well as statements about particular others, presuppose the mental representation of origos that are available for projection in the Narrative Domain through simulation processes. Thus, when narrating an event involving herself (“*I went to a party*”), the Speaker – as primary origo – selects herself-as-secondary-origo-in-the-Narrative-Domain, to which this particular stretch of discourse is to be deictically anchored (*origo allocation*) (Fricke, 2002). Alternatively, when stating that someone will do something in the future (*Linde will go to the party*), the Speaker represents this subject’s origo in the Narrative Domain, thus construing the event from this subject’s perspective or, depending on lexical choices, of a third subject’s perspective that is either made explicit (*Linde will come to John’s party*) or remains implicit (*Linde will come to the party*) (Langacker, 1987). Note that this Network is inherently recursive: once an origo in the Narrative Domain is selected as Speaker (*Linde said that John would come to the party*), a new Speech Act domain is embedded which gives rise to a new Narrative Domain with new projected and latent origos (Sanders et al., 2012); this is depicted on the left side in Figure 1.

## Deictic Navigation

The different origos of Speaker, Hearer, and (possibly) others are all potential vantage points to which the Speaker can anchor linguistic expressions. For an origo to become activated as subjective starting point in language, it must be embedded in deictic structures (Sanders and Van Krieken, 2019). The concept of *deixis* indicates “those aspects of language whose interpretation is relative to the occasion of the utterance: to the time of the utterance, and to times before and after the time of utterance; to the location of the speaker at the time of the utterance; and to the identity of the speaker and the intended audience” (Fillmore, 1966). More specifically, linguistic expressions are deictic when they acquire meaning through a connection with an origo and their interpretation depends on the coordinate system within which they function. Deixis, thus, covers all linguistic expressions that can only be understood in relation to the subjective orientation of an origo (e.g., temporal adverbs such as *today* and *next week* and demonstratives such as *this* and *that*). Note that in generic, expository utterances, this origo is non-subjective by nature; compare for instance the following headline of a Dutch news story: “*A woman from Vriezenveen has died because of the fact that she was struck by lightning*” (Sanders et al., 2012, p. 207).^3^ In this instance, the utterance is not deictically anchored to a subject of consciousness, resulting in a purely objective orientation of place (not determined), time (past related to an abstract now-point), and causality (objective relation between lightning strike and death).

Typical for narrative utterances, by contrast, is that deictic cues are essential to cognitively localize speech acts and their participants in (real or virtual) space and time, such as modeled in the DNN. Hinzen and Rosselló (2015) explicitly connect schizophrenic language to deixis in terms of pronouns, and Zimmerer et al. (2017) expand on this concept in introducing the notion of *deictic anchoring* to explain that disturbances in deictic structures in schizophrenic language reveal deviant ways in which patients relate their viewpoint to aspects in the world, including entities, events, locations, and time. Indeed, deixis is not limited to linguistic categories such as pronouns. Rather, it is the functional principle that connects all language to situations (Galbraith, 1995). Since such a connection is by definition bound to an origo as subject-of-consciousness, all perspective-taking involves deixis. The phenomenon of deixis is thus relevant throughout language; moreover, it is informative of the way a subject relates to self, other, and world.

Building on the notion of deictic anchoring as introduced by Zimmerer et al. (2017), our aim is to account for various and complex viewpoint structures in language, informed by the understanding that there are several inherently different requirements for correctly applying deixis in interaction (Sweetser, 2008). We adapt a variation of Sweetser’s (2008) requirements for building and understanding complex viewpoint structures, being (i) to have a bodily experience of one’s own present viewpoint and see the other as a viewpointed being; (ii) to project one’s present viewpoint onto others’ viewpoints in the past and future while maintaining the ability to separate these viewpoints from one another; and (iii) to integrate projected viewpoint structures into the actual here-and-now.^4^

**FIGURE 2:**
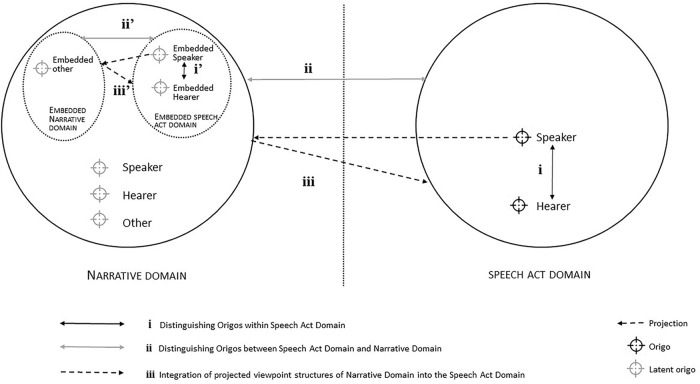
Deictic Navigation Network with three Navigation Routes: (i) the navigation of origos in the Speech Act Domain; (ii) the navigation of actual origos in the Speech Act Domain versus the navigation of projected origos in the Narrative Domain; and (iii) the integration of projected viewpoints structures in the Narrative Domain into the Speech Act Domain.

Considering recent work on the phenomenology of schizophrenia (Pienkos, 2015; Van Duppen, 2016; Fuchs and Röhricht, 2017; Ratcliffe, 2018), it is probable that in cases of schizophrenia, one or several of these requirements are not (fully) met, which is expected to be discernable in language use. On theoretical grounds, and for analytical reasons, it is important to identify exactly *how* linguistic expressions are connected to the three distinctive perspectivization requirements, i.e.: how can a study of language in schizophrenia help us understand which requirements are met and which are not and, by implication, help us gain insight into the precise nature of cognitive disturbances in perspective-taking? In the remainder of our article, we will propose that such understanding can be arrived at through application of the DNN. Figure 2 depicts the three required routes that are presupposed for successful narrative interaction. Note that each of the three routes (i–iii) has a counterpart in the embedded, recursive domains on the left side of the representation (i’–iii’).

## Linguistic Indications of Deictic Navigation Disturbances

Proposing that disturbed deictic navigation is indicative of disturbances in the three perspective-taking requirements introduced by Sweetser (2008), we will connect these requirements to the Deictic Navigation Network, coupling linguistic utterances to cognitive abilities. Our presumption is that disturbances can manifest as separate types, each indicative of a particular cognitive inability in perspective-taking with a particular (set of) linguistic counterpart(s). Presumably, however, disturbances will most often appear cumulatively, from (iii) as a higher order problem at the level of viewpoint projection and integration winding down to (i) as most fundamental.

### Speech Act Domain Disturbance (i)

Not acknowledging a bodily viewpoint that is explicitly one’s own and not seeing the other as a similarly viewpointed being reflects a radical form of self-disturbance, in that I-ness and You-ness are not adequately represented and separated, which finds its linguistic counterpart in a hybridity of subjects within the direct environment. This disturbance is located at (i) in Figure 2. The following excerpt offers an illustration:

(a) But *we* are all heroes here, aren’t *we* now? (b) If *you* think that, why don’t *you* take *my* place? (c) But then *we* never fear a place *we*’ve never been, so *you* would probably agree to do so, thinking it’s all *my* fault.^5^

In this excerpt, the Speaker merges her own origo and the Hearer’s in “we all” (sentence a) while at the same time presuming that “you,” in a conditional state (“if,” sentence b), would not, and in a generic condition (“never,” sentence c) would indeed take her place. The mixed references make it unclear whether I and You are (to be) represented as separate subjects with each their own origo and corresponding subjective orientation, or not. In addition, refraining from acknowledging one’s own and other’s (bodily) perspectives equals the negation of the concept of perspective as a whole.^6^ Therefore, a lack of epistemic expressions that moderate the attribution of feelings or perceptions to either Speaker or Hearer, and the objective, instead of subjective, construal of causal connections could also point at Speech Act Domain disturbances (Langacker, 1985; Dancygier and Sweetser, 2012). Considering the fundamental intersubjective nature of language, a breakdown of these fundamental structures of subjectivity may result in even more drastic linguistic phenomena such as privatization of language through neologisms into associative and ungrammatical discourse. Such phenomena express that the Speaker does not take into account what the Hearer requires in order to understand the Speaker’s utterances and thus linguistically alienate the Hearer’s perspective (Pienkos and Sass, 2016).

### Narrative Domain Projection Disturbance (ii)

Not being able to project origos in the Narrative domain and separate those origos from origos in the speech act domain reflects another radical disturbance. In this case, one is not able to separate I-ness here-and-now from I-ness-in-the-past/future/virtuality. This is represented by route (ii) in Figure 2. Linguistic counterparts are problems in the use of grammatical verb tense and aspect as well as temporal and spatial adverbs to anchor described events in the Narrative Domain versus the Speech Act Domain. The next excerpt gives an example.

(a) After the sentence extraordinaire (i.e., “*Love, and how important it is for everybody to love everybody*”), *came* the delusion of all delusions, the main thought that would continue to dominate my thinking for years to come. (b) The thought *was* that of a young man, the person whom I *had* last been involved with in a romantic way. (c) The sentence immediately *made* me think of my friend, who for some strange reason *became* the cause of all causes! (d) He *knew* all the answers, I thought! (e) This *was* not just some answers to some things, but all the answers about everything to do with life! (f) How *could* someone be so dumb, you say, to come to such a conclusion? (g) I *don’t* know that. (h) All I *can* say is that I *have* always been considered an intelligent person, with talents and abilities that *have been* noted by others. (i) It *was* not my intelligence, but rather my sense of religion that *might* be compared to one persuaded by a cult. (j) But in this case, my mind *came* to this conclusion all by itself. (k) Sometimes it *makes* me feel that I just *can*’t handle all of the complexities of life. (l) But up to that point I *had been* excited about life; there *were* so many new challenges in my life, and I *had* just *come* to a big city and *had fallen* in love with all it had to offer.^7^

In this excerpt, the Speaker uses present perfect (a–f, h), past (i–j), present (g,h, k), and past perfect (b, l) tense interchangeably to indicate how she felt at the onset of her illness. In doing to, she displays skill in the complex interplay of tenses that indicate different, and at times contradictory and confusing viewpoints on the time-line within the Narrative Domain. Yet, the boundary crossing between Speech Act and Narrative domain, as to where a particular stretch is (to be) represented (or in both at the same time), is not always clear. In (i), the Speaker’s origo is anchored in the Narrative Domain by “*was*,” while “*might*” anchors it in Speech Act Domain. This modal verb indicates that the Speaker is making a tentative observation, but it is unclear when and where this observation is to be represented: here-and-now, or at the time of the illness onset, or both? In addition, the boundary between Speech Act and Narrative domain crossed between sentences (j), where “*came*” projects the origo in the Narrative Domain, and (k) which is a generic utterance which is anchored by “*makes*” and “*can’t*” in the Speech Act Domain, again alternated with the Narrative Domain in (l) which is signaled by the deixis of “*that* point.”

### Speech Act and Narrative Domain Navigation Disturbance (iii)

Being unable to integrate projected viewpoints from the Narrative Domain in the actual here-and-now indicates a third radical disturbance. This action is represented in Figure 2 by route (iii) from Speech Act Domain to Narrative Domain and back. Linguistic counterparts are inappropriate use of underspecified pronouns versus overspecified nouns to refer to what is known versus new, and inappropriate use of indefinite versus definite articles (cf. the concept of deictic anchoring as proposed by Zimmerer et al. (2017). An example is provided by the following excerpt:

(a) I am a dependent on science. (b) My sanity depends on what fumbly old men can come up with in the area of discovery. (c) I could praise *him*, for already *he* has “cured” my hallucinations, but my depression prevents me. (d) You can’t be as smart as *the next man* if you haven’t had like experiences.^8^

In this excerpt, it is difficult for the Hearer to anchor the subject depicted by the Speaker in the Narrative Domain. This subject is referred to in (c) by the underspecified pronouns “him” and “he,” which makes it unclear whose origo(s) are (to be) represented in the Narrative Domain as the subject who “has cured my hallucinations” (c), since the subsequent sentence (d) does not resolve this either. As a result, the projection of the origo of “he” (i.e., the subject who has “cured” hallucinations in the past) in the Narrative Domain is hampered through the use of an underspecified pronoun, which in turn inhibits the integration of this projected viewpoint into the present in which the Speaker could praise “him” for his past deed.

## Discussion

In developing and applying the Deictic Navigation Network (DNN) to language in schizophrenia, we aim to enable the identification of localizable perspectivization disturbances, which is of importance as it could reveal and clarify fundamental disturbances in one’s ability to build complex viewpoint structures in language as well as cognition (Sweetser, 2008). DNN-analyses may show, for instance, that a patient is perfectly able to recognize and distinguish perspectives within the Speech Act Domain (route i) but, to a smaller or larger extent, fails at separating perspectives in the Speech Act Domain from perspectives represented in the Narrative Domain, indicating a difficulty in navigating between her/his own subjective consciousness and that of others, not present but projected in a Narrative Domain (route ii).^9^ Such outcomes would advance our understanding of the nature of perspective taking disturbances in schizophrenia.

Ultimately, results would shed a light on gradual differences in viewpoint taking abilities, and do justice to the rich and complicated ways in which humans navigate (linguistic) intersubjective relations. An advantage of this approach is that the DNN accounts for various inherently different types of viewpoint disturbances, which can manifest in different combinations, thus allowing for in-depth insights into the nature of perspectivization issues at the level of individuals, and of specific patient groups.

Note that each of the disturbances will amount to a fourth, radical disturbance: an incapability to represent situations from both a global and a participant viewpoint and negotiate between the two (Sweetser, 2008). When integration of general knowledge into the specific interaction in the Speech Act Domain fails, the common ground between Speaker and Hearer will be hampered, generating unfounded assumptions of general knowledge in the Hearer and manifesting itself in the unsuccessful introduction of new topics. Thus, the DNN can be applied to examine alternative disorders as well. Autistic Spectrum Disorders, for example, are characterized by impairments in perspective-taking skills (Baron-Cohen et al., 1985). Analyses of the deictic navigation abilities of people suffering from autism, such as the inability to navigate temporal expressions (Overweg et al., 2018), could shed further light on the nature of such impairments, and the way in which these abilities are conceptually connected to Theory of Mind (ToM). Deictic navigation within the Speech Act Domain corresponds to the capability of recognizing and embodying another subject’s here-and-now-present physical viewpoint, whereas deictic navigation between the Speech Act Domain and the Narrative Domain corresponds to the capacity to think about other subjects as potential subjects-of-consciousness, that is, persons who may be envisioned in terms of ‘he thinks that “p” and “he thinks that if p then q” (Goldie, 2007). Such as depicted in Figure 2, the DNN explains for this phenomenon in terms of representation in the Narrative Domain and in the embedded Speech Act Domain, respectively. Navigating the origos within and between these structures seems a crucial prerequisite for successful communicative interaction.

## Author Contributions

LvS, KvK, and JS provided substantial contributions to the conception and/or design of the work and the drafting and revising of the work. They provided approval for publication of the content and agreed to be accountable for all aspects of the work in ensuring that questions related to accuracy or integrity of any part of the work are appropriately investigated and resolved.

## Conflict of Interest Statement

The authors declare that the research was conducted in the absence of any commercial or financial relationships that could be construed as a potential conflict of interest.

## Footnotes

1 The notion of ToM is here used as an umbrella term for all “mindreading” and social cognition, and not as a commitment to Theory, Simulation Theory or Interaction Theory but to a pluralistic approach in which individuals make use of whatever procedure is cognitively least demanding to them in a given context, presuming that aside from theory and simulation, associations of behaviors with familiar agents play a crucial role in social understanding (Fiebich and Coltheart, 2015).

2 Pienkos (2015) describes four different alterations in the intersubjective experience of schizophrenic patients: (a) abnormalities of common sense and conventionality, (b) a pathological sense of openness, (c) feelings of paranoia and of centrality, and (d) perceptions of devitalization of others. Patients may, for example, experience others as a threat to their subjectivity because they experience a blurring of boundaries between themselves and whoever they interact with, i.e., a merging of their own perspective and that of the other. Alternatively, patients may construe the other as a constant threat, keeping others at a distance at all times, or even fail to recognize the other as a viewpointed being all together. These different disturbances may all together result in the (partial) loss of a shared world of (linguistic) meaning. In conversational contexts, these alterations are expected to manifest in the linguistic orchestration and navigation of the multitude of perspectives involved in interaction.

3 The defining difference between expository and narrative utterances is the representation of a subject-of-consciousness (SoC) whose origo can be projected upon as viewpoint (Sanders et al., 2012). In the news text from the example, subsequent to the expository headline, a short narrative with subjective origos unfolds: “(a) She was riding her bike on Friday morning on the Vriezenveenseweg in Almelo when the thunderstorm broke out. (b) A city doctor who arrived at the scene investigated the spot with the technical research group. (c) They came to the conclusion that the woman died due to lightning strike.” In sentence a, the deictic viewpoint is with the woman (she) as SoC, which is then moved in sentence b from herself (c: the doctor *arrived at* the scene) to the doctor and the research team as SoC (who *came to the conclusion*). Dutch: (a) Ze fietste vrijdagmorgen op de Vriezenveenseweg in Almelo toen het noodweer uitbrak. (b) Een gemeente-arts die ter plaatse kwam, heeft samen met de technische recherche de plek onderzocht. (c) Ze zijn tot de conclusie gekomen dat de vrouw is overleden door blikseminslag. Source: July 19, 2009, RTV Oost nieuwsbericht at https://www.rtvoost.nl/nieuws/99272/Vrouw-overleden-na-blikseminslag). Even in the absence of an explicit subject in the narrative discourse, some implicit SoC is necessarily represented as the observer. Compare: “(a) She was riding her bike on Friday morning on the Vriezenveenseweg in Almelo when the thunderstorm broke out. (b) The spot of the accident was at the crossing with the Almeloseweg. (c) On the women’s left, as she rested on the ground, was her purse, on her right was her bike. (d) The woman had died due to lightning strike.” In other words, the Narrative Domain *per se* has a subject-of-consciousness, who – as subject – is represented as a deictic origo by the Speaker (Sanders et al., 2012).

4 In addition, Sweetser (2008) points out that it is necessary to integrate global, less viewpointed knowledge as well as local, participant viewpoints and to meta-navigate this viewpoint system. These meta-viewpoint requirements fall beyond the scope of the present study.

5 Source: First Person Account: The Delusion Girl, Diary of a Schizophrenic, by Antoinette Rosa Ganim. In *Selected Articles from Schizophrenia Bulletin*, 13, no. 4 737-739 (1987). (United States Government Printing Office, Washington, DC, 1987) July 11, 2017 Counseling and Psychotherapy Transcripts, Client Narratives, and Reference Works https://asp6new.alexanderstreet.com/psyc/psyc.object.details.aspx?dorpID=1000048455 3/3, (p. 739)

6 Wittgensteinian analysis of the “quasi-solipsism” of psychosis, see (Sass, 1994).

7 Source: First Person Account: The Delusion Girl.

8 Source: First Person Account: The Delusion Girl.

9 The extent of such failure may be attested by quantification of such instances in the analysis of a larger corpus of utterances, such as exemplified in Van Krieken and Sanders (2019).
